# Interactions between sex and the age at disease onset on cardiometabolic risk factors in a Ghanaian population with type 2 diabetes mellitus: A cross‐sectional study

**DOI:** 10.1002/hsr2.1200

**Published:** 2023-04-13

**Authors:** Martin Awe Akilla, Moses Banyeh, Ignatius Abowini Nchor Awinibuno

**Affiliations:** ^1^ Department of Medical Laboratory Technology, School of Applied Science and Art Bolgatanga Technical University Bolgatanga Ghana; ^2^ Department of Biomedical Laboratory Science, School of Allied Health Sciences University for Development Studies Tamale Ghana; ^3^ Directorate of Allied Health, Ministry of Health Accra Ghana

**Keywords:** cardiovascular diseases, cholesterol, diabetes mellitus, fasting, LDL, type 2

## Abstract

**Background and Aim:**

The are sex differences in cardiometabolic risk factors in type 2 diabetes mellitus (T2DM) as well as the age at disease onset. However, the impact of these risk factors on the age at onset of T2DM is less known in the Ghanaian population. An understanding of the differential impact of cardiometabolic risk factors on the age at onset on T2DM may lead to sex‐specific interventions in preventive and management strategies for T2DM.

**Methods:**

The study was cross‐sectional from January to June 2019 at the Bolgatanga regional hospital. The study involved 163 T2DM patients (Female = 103, Male = 60), aged from 25 to 70 years. The body mass index (BMI) and the waist‐to‐hip ratio (WHR) were measured following standardized anthropometric techniques. Fasting venous blood samples were collected and analyzed for cardiometabolic risk factors including total cholesterol (TCHOL) and low‐density lipoprotein (LDL) cholesterol.

**Results:**

While TCHOL was higher in males than females (mean [SD]: *F* = .78 [1.37], *M* = 4.27 [1.39]) and LDL higher in females than males (mean [SD]: [*F* = 4.33 [1.22], *M* = 3.87 [1.26]), these did not, however, attain conventional statistical significance for TCHOL (*t* = 1.985, *p* = 0.05) and LDL (*t* = 2.001, *p* = 0.05). There were however, significant interactions between sex and the age at disease onset on TCHOL (*t* = −2.816, *p* = 0.006) and LDL (*t* = −2.874, *p* = 0.005), which were independent of the BMI, WHR and disease duration. The relationship between the age at disease onset and that of TCHOL and LDL were positive in females but negative in males.

**Conclusion:**

Fasting plasma TCHOL and LDL increases with increasing age at onset of T2DM in females but decreases in males. Strategies for the prevention and management of T2DM should be sex‐specific. Females with T2DM should be given more attention regarding their fasting plasma cholesterol (total) and LDL cholesterol as they are more likely than men to have increased levels of these lipids with increasing age at disease onset.

## 1 INTRODUCTION

There are sex differences in the etiology, prevalence and pathophysiology of type 2 diabetes mellitus (T2DM).^1^ It has been observed that impaired fasting glucose (IFG) is more common among men while impaired glucose tolerance (IGT) is more prevalent among women.^1^, ^2^ The transition from prediabetes to full‐blown diabetes is also shorter in men than women averaging about 8.0 and 10.0 years, respectively.^3^ There is also a sex differencein the age at disease onset and cardiometabolic risk factors associated with T2DM.^4^ While men may develop T2DM earlier and at a lower body mass index (BMI) than women, women with T2DM may have a higher BMI (about 2 kg/m^2^) than men at the time of diagnosis and this may be due to differences in body composition arising from differences in adiposity due to energy storage adaptations and hormonal influences.^5^ Premenopausal women tend to have gluteo‐femoral pattern (pear‐shaped) adiposity with more subcutaneous adipose tissue (SAT) while men usually experience truncal and abdominal pattern (apple‐shaped) adiposity with more visceral adipose tissue (VAT) which is more resistant to insulin action than SAT. Premenopausal women have been shown to have better lipid homeostasis and insulin sensitivity than men. However, when glucose tolerance is impaired, insulin resistance increases in women more than in men.^6^ After menopause women tend to experience altered body composition, poor lipid homeostasis with increasing dense atherogenic LDL cholesterol, increased VAT‐to‐SAT ratio and increased insulin resistance.^1^, ^7^

While the mechanism of sexual dimorphism in T2DM is not well understood, biological differences have been implicated. Estrogen has been suggested to have cardio‐ and athero‐protective properties with a hypolipidemic activity that upregulate LDL cholesterol receptors and also promote the activities of lipoprotein lipase.^8^, ^9^ While some studies have reported that postmenopausal women subjected to estrogen‐based hormonal therapy have experienced improved lipid homeostasis and a reduction in the incidence of T2DM, it is not a universal observation.^9^ Androgens have also been suggested to promote lipid and glucose metabolism as testosterone can be converted to estrogen by aromatization. However, testosterone may downregulate LDL receptor activity and at higher levels, may increase insulin resistance in both sexes.^10^ However, some authors have argued that sex is not strictly a binary variable and that a multiple of masculinities or femininities may converge and even interact with some other important sociodemographic and environmental variables.^6^ Moreover, the sex differences in cardiometabolic risk factors may not be just biological but rather due to the observation that the magnitude of deterioration before the development of T2DM is greater in women than men and this may make cardiometabolic risk factors for T2DM more pronounced in women than men.^10^ It is however, worth noting that the sex‐specific impact of cadiometabolic risk factors in T2DM onset may be modified by life‐style modifications such as diet, physical activity, smooking and alcohol consumption.^11^ There are pieces of evidence supporting the vital role of physical activity in the prevention and treatment of diabetes. Physical activity is recognized to produce multiple general and diabetes‐specific health benefits.^12^, ^13^ Also, dietary modification has the potential to appreciably reduce the risk of progression of prediabetes to T2DM. High‐profile randomized controlled trials conducted in persons of varying ethnicities in different countries have consistently shown a halving of the risk of T2DM with dietary modification.^11^, ^13^ Compliance with dietary advice results in improvement in glycemic control and reduction in cardiovascular risk regardless of duration of disease.^11^

Studies regarding the risk factors associated with T2DM in Ghana are many.^14^, ^15^ The frequency of increased insulin resistance, dyslipidemia, hypertension and altered liver and renal parameters are higher among Ghanaians with T2DM than in controls.^16^, ^17^, ^18^ However, most of these previous studies fell short of investigating the possible interactions between sex and the age at onset of T2DM. The determination of sex‐specific differences and variable interactions in T2DM is vital for strategies in the early diagnosis, therapy and management of T2DM as these interventions need to be tailored according to one's sex or the onset of T2DM or both.^6^ Since the pathophysiology of T2DM is dynamic given to genetic and environmental variabilities, there is always the need for population‐specific studies. The study aimed to determine how sex may moderate cardiometabolic risk factors of T2DM through its interactions with the age at disease onset.

## 2 MATERIAL AND METHODS

### 2.1 Study design and setting

This was a cross‐sectional study from January to December 2019 at the Bolgatanga Regional Hospital (BRH). Bolgatanga is the administrative capital of the Upper East Region (UER) of Ghana. The UER shares a border with the Northern Region to the south, The Upper West Region to the West, Burkina Faso to the North and Togo to the East. The BRH is a secondary‐level health facility in the Ghana Health Service and it is the main referral hospital in the region. The catchment area of the BRH extends beyond the UER to parts of Togo and Burkina Faso.

### 2.2 Participants and sampling

The study involved 163 T2DM patients (*F* = 103, *M* = 60), aged from 25 to 70 years. The T2DM participants were on follow‐up appointments at the diabetic clinic and were receiving metformin‐Glimepiride oral therapy based on the Ghana Health Service Treatment Protocol for T2DM.^19^ Persons with known type 1 diabetes, maturity‐onset diabetes of the young, gestational diabetes, chronic liver disease, chronic renal disease and/or hypertension were excluded. T2DM was defined per the criteria proposed by the World Health Organization Diabetes Experts Committee as a fasting blood glucose level ≥ 7.0 mmol/L or a 2‐h postload glucose ≥ 11.1 mmol/L.^20^ The duration of T2DM was defined as equaling the time from diagnosis of T2DM.^21^

### 2.3 Sample size calculation

The crude prevalence of type 2 diabetes in Ghana has been estimated to be 6.3%.^22^ Using a confidence interval (CI) of 95%, a z score of 1.96 for 95% CI, and a margin of error of 5%, the minimum sample size was estimated to be 139 using the Cohran formula^23^:

$$n=\frac{z2pq}{d2}$$

Where:

*Z* = z‐score at 95% CI

*p* = prevalence

*q* = 1‐p

*d* = margin of error

### 2.4 Variables

The dependent variables are total cholesterol (TCHOL), high‐density lipoprotein (HDL), low‐density lipoprotein (LDL), triglycerides (TRIG), and systolic blood pressure (SBP), diastolic blood pressure (DBP), and the homeostatic model assessment‐insulin resistance (HOMA‐IR). The independent variables were sex and age at T2DM onset. Confounding variables included the age at the time of sampling, disease duration, waist‐to‐hip ratio (WHR) and BMI.

### 2.5 Data sources/measurements

The sociodemographic data were collected using a pretested questionnaire. The body weight was measured when the participant was barefooted and in light clothing. Body weight was measured to the nearest 0.1 kg using a bathroom weighing scale. The standing height was measured using a stadiometer with the participant being barefooted and facing forward in the Frankfurt plane. The heels, buttocks, shoulder and occiput, touched the measuring board. The head plate was then adjusted to touch the head and the height was measured to the nearest 0.1 cm. The BMI (Kg/m^2^) was derived by dividing the body weight (Kg) by the standing height (m^2^).^24^ Blood pressure was measured from the left upper arm by a trained health professional with a mercurial sphygmomanometer. The measurement was taken after the participant had rested in a sitting position for at least 5 min and was then repeated after 5 min. The SBP and DBP were recorded in mmHg and then averaged.^25^ A single venous blood sample was collected between 8 am and 10 am after an overnight fast (12 h) into fluoride oxalate, K_3_EDTA and gel separator vacutainer tubes. The blood in the gel separator tube was allowed to clot at 4°C for 30 min before all the tubes were spun at 1500 rpm for 10 min to obtain plasma and serum respectively. The serum/plasma was aliquoted into cryovials and then stored at −25°C for later analysis. The serum/plasma samples were retrieved and thawed without refreezing and homogenized thoroughly before biochemical analysis. Serum/plasma glucose, lipids and electrolytes were measured on the BT 1500 automated analyzer (Biotechnica Instruments, SPA) using the recommended reagents and following the manufacturer's instructions. The serum/plasma insulin was measured in duplicates using ELISA test kits (Monobind Inc.). The plasma lipids were measured on the BT 1500 automated biochemistry analyzer (Biotechnica Instruments, SPA) following the manufacturer's instructions and using the recommended reagents. The HOMA‐IR was calculated using the formula below where FBG is fasting blood glucose in mmol/L and insulin in mIU/L.^26^

$$\text{HOMA}‐\text{IR}=\frac{\text{FBG}x\;\text{insulin}}{22.5}$$

### 2.6 Statistical analysis

The data were collected and analyzed in SPSS (v26) and GraphPad Prism (v8). The data were checked for normality and the presence of extreme outliers using the Shapiro‐Wilk test. Extreme values less than 5% of the data per variable were removed and then replaced with the series mean of the variable. Descriptive statistics were then performed for each variable and were presented as mean ± standard deviation (SD) or median (IQR) for parametric and non‐parametric variables respectively. The differences between male and female means or medians were tested using the Student *t*‐test or the Man−Whitney *U* tests respectively. The possible interactions between sex and the age at onset of T2DM was investigated. The age at disease onset was first centered on its mean by substracting the mean from the variable. Mean‐centering was done to reduce homoscedasticity. Two‐way interaction terms were then created between sex and centered age at onset variables (Sex*Age at onset‐centered). Linear regression models were formulated by entering sex, age at diseases onset‐centered and the two‐way interaction term as predictors while cardioembolic risk factors were the dependent variables. To reduce confounding, the age at the time of sampling, the WHR and the BMI were also entered into the model as covariates. The assumptions of linear regression of the models of interest were tested using the standardized predicted and residual values of the dependent variables. Multivariable normality was tested using the probability−probability plots (P−P plots) while a scatter plot was used to test homoscedasticity. The unstandardized predicted values of the dependent variable were saved and then plotted on the Y‐axis against the centered age at diseases onset variable on the X‐axis. Sex was then used as the marking variable to show the sex differences in the relationship between the age at disease onset and cardiometabolic risk factors. All the statistical analyses were 2‐tailed and were considered as significant at *p* < 0.050.

### 2.7 Ethical statement

The study was conducted following recommended guidelines as contained in the 1964 Declaration of Helsinki and its later amendments regarding human subject studies. The study received approval from the institutional review board of the Navrongo Health Research Center (Ref#: NHRCIRB216). Written informed consent was obtained from each participant before the study.

## 3 RESULTS

### 3.1 General characteristics of the study population

The general characteristics of the study population are summarized in Table 1. Males and females were matched by the age at the time of sampling, age at disease onset and the duration of T2DM. While, the fasting plasma TCHOL (*t* = 1.985, *p* = 0.05) was higher in males than females and LDL cholesterol (*t* = 2.001, *p* = 0.05) was higher in females than males, these did not however, attain conventional statistical significance (*p* < 0.05).

**Table 1:** The general characteristics of the study population.

Variable	Female	Male	*t‐*statistic/Mann−Witney *U*	*p* Value
Age at sampling (years)	51.5 (8.4)	53.4 (10.4)	−1.119	0.27
Age at onset (years)	47.9 (8.9)	49.6 (10.5)	−0.996	0.32
Disease duration (years)	3.0 (3.0−5.0)	3.5 (0.3−6.0)	1946.5	>0.99
BMI (Kg/m^2^)	29.2 (4.9)	24.8 (4.5)	4.884	<0.001
WHR	0.93 (0.06)	0.95 (0.08)	−1.377	0.17
TCHOL (mmol/L)	1.78 (1.37)	4.27 (1.39)	1.985	0.05
HDL (mmol/L)	1.51 (0.58)	1.43 (0.64)	0.652	0.52
LDL (mmol/L)	4.33 (1.22)	3.87 (1.26)	2.001	0.05
TRIG (mmol/L)	1.48 (0.91)	1.51 (0.89)	−0.176	0.86
SBP (mmHg)	141 (21)	136 (21)	1.358	0.18
DBP (mmHg)	90 (12)	86 (14)	1.335	0.18
HOMA‐IR (mUI/mL)	2.93 (1.52)	2.95 (1.66)	1835.0	0.94

*Note*: The results were summarized as means (SD) for parametric variables or median (IQR) for nonparametric variables. Sex differences in mean and median values were compared using the Student *t*‐test and the Mann−Whitney *U* test (unpaired, 2‐tailed) respectively.

Abbreviations: BMI, body mass index; DBP, diastolic blood pressure; HDL, high‐density lipoprotein; HOMA‐IR, homeostatic model assessment‐insulin resistance; LDL, low‐density lipoprotein; SBP, systolic blood pressure; TCHOL, total cholesterol; TRIG, triglycerides; WHR, waist‐to‐hip ratio.

### 3.2 Interactions between sex and age at onset of T2DM

**Figure 1:**
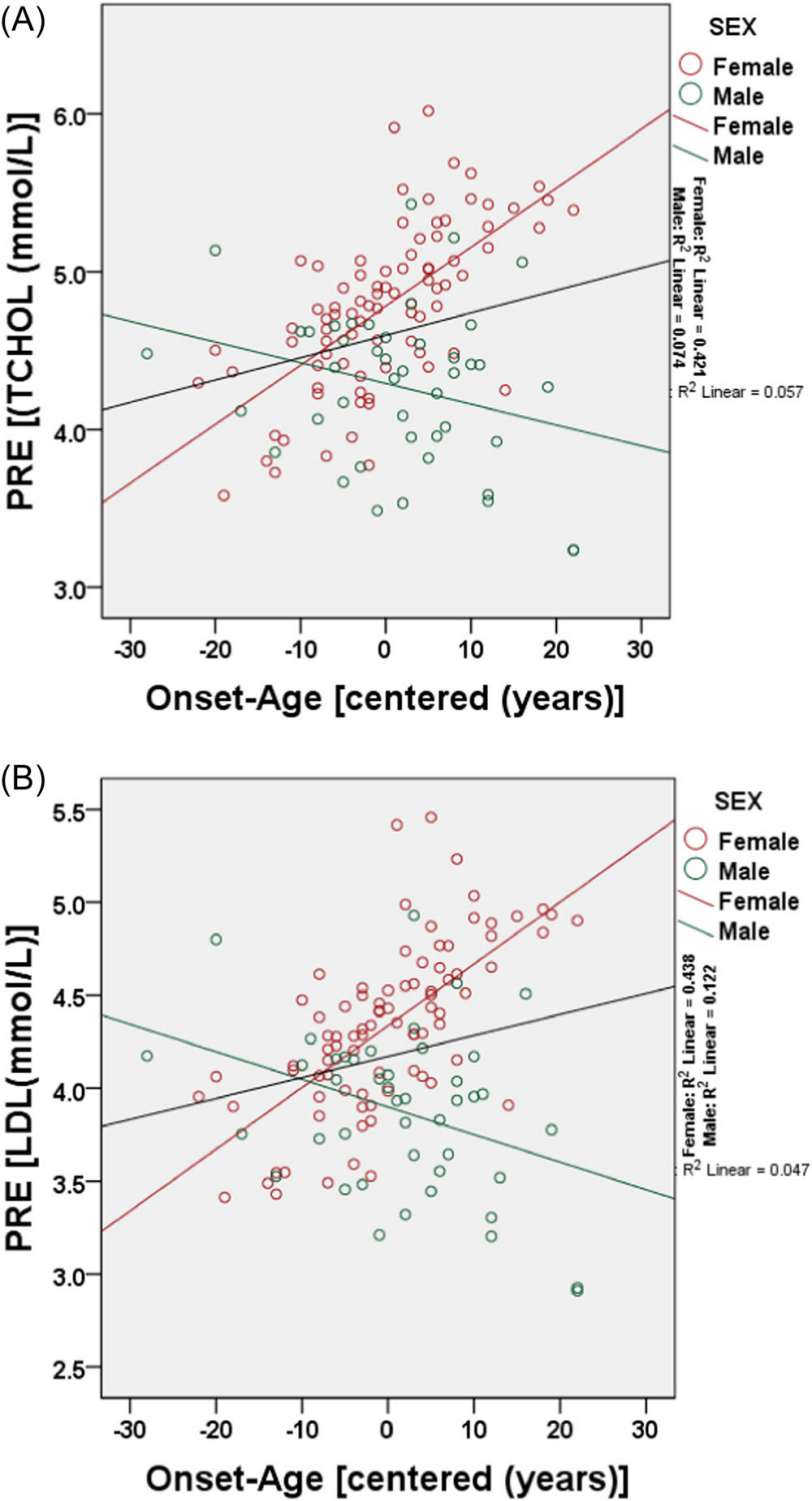
The sex‐moderated relationship between the age at disease onset and that of total cholesterol (A: TCHOL) and low‐density lipoprotein (B: LDL) cholesterol. The unstandardized predicted value (PRE) of the dependent variables (TCHOL and LDL) was plotted on the y‐axis against the mean‐centered predictor variable (onset‐age) on the x‐axis while sex was used as the marking variable.

The linear regression models with interaction terms showing the sex‐moderated relationships between the age at onset of T2DM and cardiometabolic risk factors are summarized in Table 2 and Figure 1. There were significant interactions between sex and the age at onset of T2DM on TCHOL (*p* = 0.006) and LDL (*p* = 0.005). The interactions were independent of BMI, WHR and disease duration. While TCHOL and LDL levels increased with increasing age at disease onset in females, the reverse was true in males (Figure 1). The assumptions of linear regression were tested for TCHOL and LDL using the regressions' standardized predicted and residual values (Supporting Information: Figure S1). The assumptions of normality and homoscedasticity were not violated.

**Table 2:** The sex‐moderated relationship between age at onset of type 2 diabetes on cardiometabolic risk factors.

LR	Dependent variable	*B*	95% CI		*t‐*statistic	*p* Value
			Lower	Upper		
1	TCHOL (mmol/L)					
	(Constant)	0.029	−3.279	3.338	0.018	0.99
	BMI (Kg/m^2^)	0.067	0.017	0.117	2.676	0.008
	WHR	2.829	−0.724	6.381	1.576	0.12
	Disease duration (years)	0.047	−0.022	0.115	1.349	0.18
	Sex	−0.235	−0.774	0.305	−0.862	0.39
	Onset‐age	0.047	0.014	0.080	2.819	0.006
	Sex*Onset‐age	−0.073	−0.124	−0.022	−2.816	0.006
2	HDL (mmol/L)					
	(Constant)	1.009	−0.526	2.543	1.301	0.20
	BMI (Kg/m^2^)	0.016	−0.007	0.039	1.400	0.16
	WHR	0.032	−1.615	1.680	0.039	0.97
	Disease duration (years)	−0.001	−0.033	0.031	−0.052	0.96
	Sex	0.018	−0.233	0.268	0.140	0.89
	Onset‐age	0.001	−0.015	0.016	0.075	0.94
	Sex*Onset‐age	−0.013	−0.036	0.011	−1.070	0.27
3	LDL (mmol/L)					
	(Constant)	0.577	−2.396	3.549	0.384	0.70
	BMI (Kg/m^2^)	0.057	0.012	0.101	2.508	0.01
	WHR	2.067	−1.124	5.259	1.282	0.20
	Disease duration (years)	0.053	−0.008	0.115	1.711	0.09
	Sex	−0.222	−0.706	0.263	−0.905	0.37
	Onset‐Age	0.043	0.013	0.073	2.861	0.005
	Sex*Onset‐Age	−0.067	−0.113	−0.021	−2.874	0.005
4	TRIG (mmol/L)					
	(Constant)	−1.047	−3.283	1.190	−0.926	0.36
	BMI (Kg/m^2^)	0.020	−0.014	0.053	1.150	0.25
	WHR	2.149	−0.252	4.551	1.772	0.08
	Disease duration (years)	−0.012	−0.058	0.035	−0.503	0.62
	Sex	0.057	−0.308	0.422	0.309	0.76
	Onset‐age	0.019	−0.004	0.041	1.663	0.10
	Sex*Onset‐age	−0.007	−0.042	0.027	−0.409	0.68
5	SBP (mmHg)					
	(Constant)	146.017	94.200	197.835	5.578	<0.001
	BMI (Kg/m^2^)	0.322	−0.455	1.100	0.820	0.41
	WHR	−17.959	−73.597	37.678	−0.639	0.52
	Disease duration (years)	0.637	−0.435	1.709	1.176	0.24
	Sex	−5.051	−13.502	3.400	−1.183	0.24
	Onset‐age	0.808	0.289	1.327	3.080	0.003
	Sex*Onset‐age	−0.067	−0.867	0.733	−0.166	0.87
6	DBP (mmHg)					
	(Constant)	79.393	46.961	111.825	4.846	<0.001
	BMI (Kg/m^2^)	0.362	−0.124	0.849	1.474	0.14
	WHR	−1.997	−36.820	32.826	−0.114	0.91
	Disease duration (years)	0.400	−0.270	1.071	1.181	0.24
	Sex	−1.965	−7.255	3.324	−0.736	0.46
	Onset‐age	0.220	−0.106	0.545	1.337	0.18
	Sex*Onset‐age	−0.024	−0.525	0.477	−0.095	0.92
7	HOMA‐IR (mUI/mL)					
	(Constant)	3.315	−0.579	7.209	1.685	0.10
	BMI (Kg/m^2^)	−0.002	−0.060	0.057	−0.064	0.95
	WHR	−0.782	−4.963	3.399	−0.370	0.71
	Disease duration (years)	0.111	0.031	0.192	2.731	0.007
	Sex	0.061	−0.574	0.696	0.190	0.85
	Onset‐age	−0.003	−0.042	0.036	−0.136	0.89
	Sex*Onset‐age	−0.027	−0.087	0.033	−0.878	0.38

*Note*: The onset age was centered on its mean before analysis. The models were adjusted for BMI, WHR and disease duration.

Abbreviations: BMI, body mass index; DBP, diastolic blood pressure; HDL, high‐density lipoprotein; HOMA‐IR, homeostatic model assessment‐insulin resistance; LDL, low‐density lipoprotein; SBP, systolic blood pressure; TCHOL, total cholesterol; TRIG, triglycerides; WHR, waist‐to‐hip ratio.

## 4 DISCUSSION

The study aimed to determine the possible interactions between sex and the age at onset of T2DM on cardiometabolic risk factors. The TCHOL was higher in males while LDL was higher in females but the difference did meet conventional statistical significance. There were significant interactions between sex and the age at onset of T2DM on fasting plasma TCHOL and LDL. While TCHOL and LDL increased with increasing age at disease onset in females, the reveres was observed in males.

The fasting plasma TCHOL was higher in males while the LDL was higher in females, albeit not statistically significant. Lipids generally reflect a person's lifestyle, genetics and protein variables which may cumulatively influence insulin resistance and the onset of T2DM.^27^ When insulin binds to the insulin receptor, in healthy subjects, the hormone‐sensitive lipase (HSL), which is involved in the hydrolysis of TRIG in adipocytes, is inactivated. However, in a state of insulin resistance, the activity of HSL increases resulting in increased levels of free fatty acids from triglyceride hydrolysis which are then picked up by hepatocytes and channeled into other secretory pathways involved in the production of LDL. The free fatty acids interfere with the insulin signaling pathway by increasing the levels of reactive oxygen species, diacylglycerol and protein kinase C. There are further alterations in the phosphorylation of insulin receptor substrate‐1 (IRS‐1) which then affect Beta‐cell function with further insulin resistance, metabolic dysregulation and finally T2DM.

However, previous studies have observed that cholesterol metabolism is sex‐dependent. Premenopausal women tend to have higher VLDL and LDL production and hydrolysis than men. But premenopausal women have better lipid homeostasis than similarly aged men as the transport and removal of LDL is more efficient in women due to their increased lipoprotein lipase activity. It has been suggested that estrogen upregulates while androgens inhibit the activity of LDL receptors.^10^ This results in premenopausal women having low LDL than men.^2^, ^28^ This special adaptation differentially protects premenopausal women from hypercholesterolemia and other metabolic diseases but not after menopause. The phenomenon is not well understood but has been attributed to the hypolipidemic effect of estrogens.^8^, ^9^ After menopause, however, there is altered lipid homeostasis and the accumulation of LDL due to the decline in estrogen levels. Insulin resistance further alters lipid metabolism resulting in increased plasma levels of LDL in women.^10^, ^29^ Better lipid homeostasis in premenopausal women may explain the findings in this study of higher TCHOL and LDL, in early than late‐onset T2DM in men and vice‐versa in women. Women with early‐onset T2DM may still have relatively higher estrogen than women with late‐onset T2DM. But since androgens may impact insulin resistance, men with early‐onset T2DM may have poorer lipid homeostasis and will tend to improve as androgens levels decline with age. The positive effect of estrogen on lipid homeostasis is not always observed among postmenopausal women or women with the polycystic ovarian syndrome who underwent estrogen therapy.^9^ Also, some authors have argued that sex differences in cardiometabolic risk factors may not be biological but rather due to the observation that women undergo a greater cardiometabolic risk deterioration before the onset of T2DM than males. It has also been suggested that IGT, which is more common in women than men may be associated more with increased cardiovascular risk and progression to diabetes than IFG. While IFG may be due to impaired early insulin secretion and increased hepatic glucose output, IGT is mainly due to peripheral insulin resistance.^6^

This study is the first to demonstrate that there are interactions between sex and the age at onset of disease among a T2DM patient population in Northern Ghana. In this study, the assumptions of linear regression were tested and were shown not to have been violated. The regression models were adjusted for confounding variables such as BMI and WHR which could potentially bias the outcome. Although metformin‐glimepiride therapy may have a lipid‐lowering effect in T2DM, this did not mask the sex differences in lipid homeostasis in the study population. It may appear the sample size was small, however, the number of participants was based on the prevalence of T2DM in Ghana.^22^ The study was however, limited in the following way: The onset of T2DM is significantly affected by life‐style modications such as diet, smooking and physical exercise.^12^, ^13^ However, these variables were not considered in the present study. Family history of T2DM is also a significant risk factor as there are candidate genetic variants that are associated with T2DM.^30^ It is recommended that future studies should consider family history, life‐style and dietary habits in the investigation of risk factors of T2DM.

## 5 CONCLUSION

There are significant interactions between sex and age at disease onset among T2DM patients in Ghana. There are sex differences in the relationships between fasting plasma TCHOL, LDL cholesterol and the age at disease onset in T2DM. The treatment for lipid control among T2DM patients should be sex‐specific with females requiring further intervention than males, particularly in late‐onset T2DM.

## AUTHOR CONTRIBUTIONS

**Martin Awe Akilla**: Conceptualization; data curation; investigation; methodology; writing—review and editing. **Moses Banyeh**: Formal analysis; writing—original draft; writing—review and editing. **Ignatius Abowini Nchor Awinibuno**: Data curation; investigation; writing—review and editing.

## CONFLICT OF INTEREST STATEMENT

The authors declare no conflict of interest.

## TRANSPARENCY STATEMENT

The lead author Moses Banyeh affirms that this manuscript is an honest, accurate, and transparent account of the study being reported; that no important aspects of the study have been omitted; and that any discrepancies from the study as planned (and, if relevant, registered) have been explained.

## Supporting information

Supporting information.

## Data Availability

The data supporting the findings in this study will be made available through the corresponding author upon reasonable request. The corresponding author had full access to all of the data in this study and takes complete responsibility for the integrity of the data and the accuracy of the data analysis.
