# Supplementary material for: Interactions between sex and the age at disease onset on cardiometabolic risk factors in a Ghanaian population with type 2 diabetes mellitus: A cross‐sectional study
Source: Health Sci Rep. 2023 Apr 13;6(4):e1200. doi: 10.1002/hsr2.1200 (PMC10098444; doi:10.1002/hsr2.1200)
Supplement: Supplementary file 1 — Supporting information. [file HSR2-6-e1200-s001.docx]

**Supplementary file**

**Supplementary Figure S1.** A test of the assumptions of linear regression for total cholesterol (TCHOL) and low-density lipoprotein cholesterol (LDL). The standardized (Z) regression predicted values and residuals of the dependent variables were plotted on the y- and x-axis respectively. Multivariable normality was tested using a histogram (Top) and Probability-Probability plot (middle), while homoscedasticity was tested using a scatter plot (bottom).
